# Recurrent venous thromboembolism despite natural cycle frozen embryo transfer in a woman with a history of pregnancy-related venous thromboembolism: A case report

**DOI:** 10.20407/fmj.2026-002

**Published:** 2026-09-25

**Authors:** Yutaro Sato, Eiji Nishio, Shota Oikawa, Sana Odake, Miho Ishikawa, Eriko Sakakibara, Kiriko Kotani, Hikari Yoshizawa, Hironori Miyamura, Haruki Nishizawa, Iori Kisu

**Affiliations:** Department of Obstetrics and Gynecology, Fujita Health University, School of Medicine, Toyoake, Aichi, Japan

**Keywords:** Venous thromboembolism, Pregnancy, Frozen embryo transfer, Inferior vena cava filter, Hypercoagulable state

## Abstract

Pregnancy-associated venous thromboembolism (VTE) is a major cause of maternal morbidity and mortality. Pregnancies achieved by *in vitro* fertilization carry an increased risk of VTE, particularly during early gestation. A 33-year-old woman developed deep vein thrombosis during late pregnancy in her first spontaneous conception despite no identifiable thrombophilia. During a subsequent pregnancy achieved by natural cycle frozen embryo transfer, which was selected to avoid exogenous estrogen exposure and potentially reduce the thrombotic risk compared with hormone replacement cycles, she developed recurrent VTE at 8 weeks of gestation. Anticoagulation therapy was initiated, and an inferior vena cava (IVC) filter was placed in late pregnancy because of persistent thrombus and delivery-related risk. A cesarean section was successfully performed, and the IVC filter was removed postpartum after regression of the thrombus. Although natural cycle frozen embryo transfer may reduce the thrombotic risk compared with hormone replacement cycles, it does not eliminate the risk of recurrent VTE in women with a history of pregnancy-associated thrombosis. Early initiation of anticoagulation and careful peripartum management, including selective use of IVC filters, are essential in high-risk patients.

## Introduction

Venous thromboembolism (VTE) occurs in 1 to 2 of every 1,000 pregnant women during pregnancy or postpartum.^1^ A pulmonary embolism is the leading cause of maternal death. Therefore, managing pregnancy requires a thorough understanding of the risk of developing VTE and how to manage it. The risk of VTE in *in vitro* fertilization (IVF) pregnancies is twice that of spontaneous pregnancies, increasing 5- to 10-fold, especially in the first trimester.^2^ This increase is due to the high estrogen state induced by ovulation-inducing agents, which enhance blood coagulation. Furthermore, the incidence of VTE considerably increases in IVF pregnancies complicated by ovarian hyperstimulation syndrome, with some reports indicating a rate approximately 100 times higher than that in natural pregnancies.^2^ Furthermore, the hormone replacement cycle involves the administration of estrogen and progesterone, which makes it prone to hypercoagulability.

Consequently, the risk of thrombosis during hormone replacement cycle embryo transfer is higher than that during natural cycle frozen embryo transfer.^3^ The primary treatment for VTE during pregnancy is anticoagulation therapy using low-molecular-weight heparin. Filter placement should only be considered in exceptional circumstances, such as when anticoagulation therapy is contraindicated or ineffective.^4,5^

We present the case of a patient who developed VTE during her first pregnancy, despite having no apparent coagulation abnormalities, and who subsequently desired a second pregnancy. Natural cycle frozen embryo transfer (NC-FET) was performed to reduce thrombotic risk; however, VTE recurred at 8 weeks of gestation. An inferior vena cava (IVC) filter was then placed at 35 weeks and 0 days of gestation, and a cesarean section was performed.

## Case presentation

A 33-year-old woman conceived her first child spontaneously and developed lower extremity venous thrombosis at 31 weeks of gestation. She underwent IVC filter placement and continuous intravenous heparin infusion. She delivered a 2,456 g neonate via cesarean section at 37 weeks of gestation. After giving birth, she took warfarin for 4 months. Blood samples were collected after the first delivery to screen for coagulation abnormalities, but none were found (protein C antigen level: 127%; protein S antigen level: 75%; lupus anticoagulant: 1.0; anti-CL/βGPI antibody: 1.2 U/mL; anti-cardiolipin antibody: 4.0 U/mL).

The couple wanted a second child but were unable to conceive naturally. Therefore, they underwent IVF and the blastocyst was frozen on day 5 after fertilization, following egg retrieval. Six months later, following a frozen blastocyst transfer, NC-FET was performed to avoid exogenous estrogen exposure and reduce the thrombotic risk compared with hormone replacement cycles, resulting in a successful pregnancy. Although the patient had a history of pregnancy-associated VTE, prophylactic anticoagulation was not initiated immediately after confirming pregnancy because no thrombophilia had been identified, the previous thrombotic event had occurred in late pregnancy, and NC-FET was selected to reduce the hormone-related thrombotic risk.

She presented at 8 weeks of gestation with pain in her left lower limb. Swelling was observed in the left lower limb. An ultrasonographic examination of the lower extremities showed a thrombus in the left soleus vein. Her serum D-dimer level was 7.6 μg/mL. She was administered 10,000 units of heparin calcium subcutaneously daily for thromboprophylaxis. She was initially treated as an outpatient but was admitted at 34 weeks and 5 days of gestation because of warning signs of bleeding caused by a low-lying placenta. Based on the fact that the residual thrombus was confirmed before the caesarean section, delivery was considered to carry a high risk of pulmonary embolism because of hemodynamic changes and release of vena caval compression during caesarean section. An IVC filter was placed at 35 weeks.

A cesarean section was performed at 35 weeks and 1 day, resulting in the delivery of a 1,915 g neonate. A computed tomography scan of the lower extremities revealed a thrombus in the left soleus vein (Figure 1A). The IVC filter was scheduled for removal on the first postoperative day, but this was cancelled after a contrast-enhanced computed tomography scan showed that the filter was attached to a thrombus (Figure 1B). Therefore, heparin administration was resumed. A follow-up ultrasonographic examination of the lower extremities confirmed a reduction in the thrombus, allowing for IVC filter removal on postoperative day 13.

## Discussion

There are two notable implications of our case. First, despite performing an embryo transfer after natural ovulation to reduce the risk of VTE, VTE recurred at 8 weeks of pregnancy Therefore, although NC-FET may reduce the thrombotic risk compared with hormone replacement cycles, it cannot completely prevent recurrent VTE in high-risk patients. Second, placement of an IVC filter could be an option for preventing pulmonary embolisms.

Changes in hemodynamics caused by hormonal changes are thought to be the cause of VTE occurring in early pregnancy. Consistent with previous reports,^6^ VTE developed at 31 weeks during the first pregnancy and at 8 weeks during the second pregnancy in our case. Baba et al. found that the median gestational age at the time of VTE onset was 31 weeks.^6^ In their study, the incidence of VTE showed a bimodal distribution where 126 (30.7%) women developed VTE in the first trimester (before 14 weeks of gestation) and 236 (57.6%) developed VTE in the third trimester (after 28 weeks). This distribution may be caused by underlying complications, such as dehydration due to hyperemesis gravidarum, prolonged bed rest, pregnancy-induced hypertension, placental abnormalities, and compression of the inferior vena cava by the uterus. The recurrence rate within 10 years of the onset of pregnancy-related VTE is approximately 13%, but the cumulative risk subsequently increases. Reports have indicated that approximately 30%–40% of cases experience recurrence of VTE after 30 years.^7^ Therefore, careful long-term follow-up and anticoagulant therapy to prevent recurrence should be considered for women with a history of pregnancy-related VTE. One reported reason for the increased procoagulant state during hormone replacement cycles is the considerable increase in thrombin generation due to estrogen administration.^8^ In this case, despite undergoing NC-FET, the patient developed VTE during early pregnancy. This finding suggests that although NC-FET may reduce the thrombotic risk compared with hormone replacement cycles, it cannot completely prevent recurrence of VTE in high-risk patients.

The recent American Society of Hematology guideline on the management of venous thromboembolism in pregnant women does not address the issue of whether to insert a IVC filter to treat acute VTE in the third trimester of pregnancy.^9^ The older American College of Chest Physicians guideline discusses the use of IVC filters, restricting them to women at high risk of recurrence, such as those with proven deep vein thrombosis and recurrent pulmonary embolism despite anticoagulant treatment.^10^ Some reports have indicated that the risks of filter placement during pregnancy are comparable to those in non-pregnant cases.^4^ However, another study reported that at least one filter-related complication was reported in 33 of 177 (19%) women, including in-filter thrombosis (14 cases), tilt (6 cases), migration (5 cases), perforation (2 cases), fracture (3 cases), misplacement (1 case), air embolism (1 case) and an allergic reaction (1 case).^5^ Two (1%) filter complications led to maternal death, and at least one of them was directly associated with filter insertion. Filter retrieval failed in nine (6%) women.^5^ Therefore, the advantages and disadvantages of filter placement remain controversial, and the use of IVC filters during pregnancy should be carefully evaluated.

In this case, anticoagulant therapy was initiated in the first and second pregnancies. However, residual thrombus was confirmed before caesarean section. An IVC filter was inserted because cesarean delivery was considered to have a high risk of pulmonary embolism owing to hemodynamic changes and release of vena caval compression. A contrast-enhanced computed tomography scan after the second cesarean section showed a clot trapped within the filter despite the patient’s ongoing anticoagulant therapy. Whether this thrombus formed within the filter or was a free-floating clot that became trapped is unclear. Nevertheless, this finding suggested that a high thrombotic risk persisted even after terminating pregnancy and during anticoagulant therapy.

In summary, in infertile patients with a history of VTE during pregnancy, NC-FET may reduce the thrombotic risk compared with hormone replacement cycles but does not eliminate the risk of recurrence VTE. Prophylactic anticoagulation therapy must be initiated immediately upon confirming the pregnancy and requires long-term management.

## Figures and Tables

**Figure 1:**
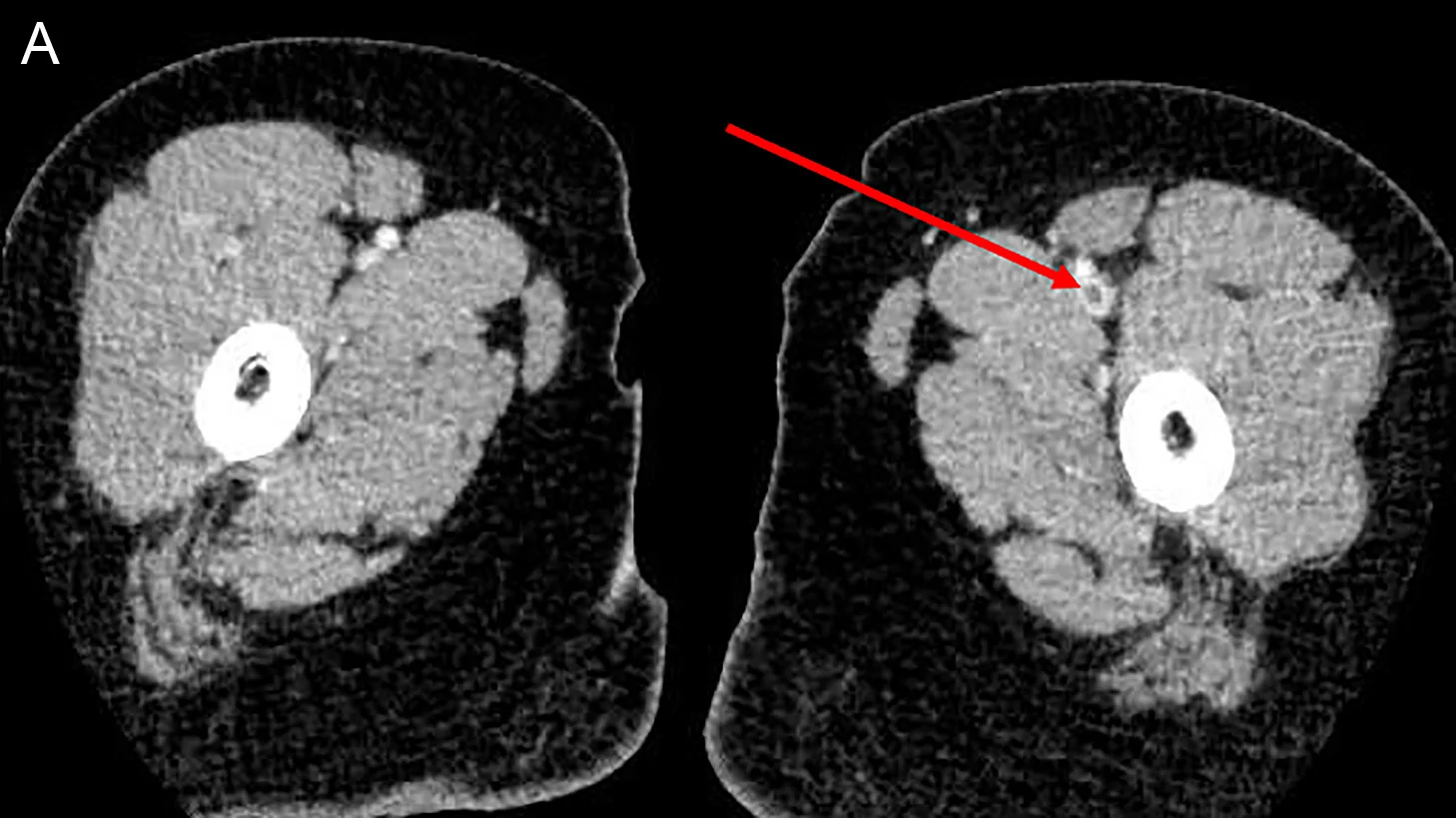

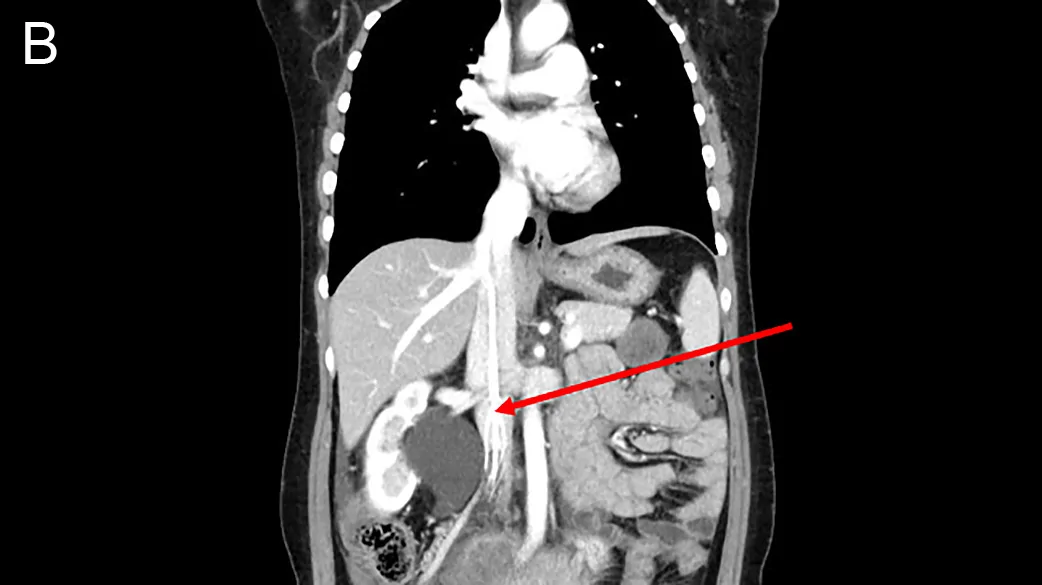
A: Left lower extremity deep vein thrombosis (arrow). B: Inferior vena cava filter (arrow).
